# Interplay of Dynamic Transcription and Chromatin Remodeling: Lessons from Yeast

**DOI:** 10.3390/ijms12084758

**Published:** 2011-07-25

**Authors:** Gerhard Niederacher, Eva Klopf, Christoph Schüller

**Affiliations:** 1 Max F. Perutz Laboratories, Department of Biochemistry and Cell Biology, University of Vienna, 1030 Vienna, Austria; E-Mails: gerhard.niederacher@univie.ac.at (G.N.); eva.klopf@univie.ac.at (E.K.); 2 Department of Applied Genetics and Cell Biology, University of Natural Resources and Life Sciences, Vienna, UFT Campus Tulln, 3430 Tulln, Austria

**Keywords:** chromatin, *S. cerevisiae*, stress, transcription

## Abstract

Regulation of transcription involves dynamic rearrangements of chromatin structure. The budding yeast *Saccharomyces cerevisiae* has a variety of highly conserved factors necessary for these reconstructions. Chromatin remodelers, histone modifiers and histone chaperones directly associate to promoters and open reading frames of exposed genes and facilitate activation and repression of transcription. We compare two distinct patterns of induced transcription: Sustained transcribed genes switch to an activated state where they remain as long as the induction signal is present. In contrast, single pulsed transcribed genes show a quick and strong induction pulse resulting in high transcript levels followed by adaptation and repression to basal levels. We discuss intensively studied promoters and coding regions from both groups for their co-factor requirements during transcription. Interplay between chromatin restructuring factors and dynamic transcription is highly variable and locus dependent.

## 1. Introduction

Eukaryotic DNA is complexed with histone octamers, which are composed of dimers of the core histones H2A, H2B, H3 and H4. 147 bp of DNA are wrapped 1.65 times around each octamer forming nucleosomes, the basic packaging units of chromatin [1]. Nucleosomes, connected by linker DNA of variable length as “beads on a string”, generate the 11 nm linear structure. The linker histone H1 is positioned at the top of the core histone octamer and enables higher organized compaction of DNA into transcriptionally inactive 30 nm fibres. In addition to topological DNA compaction chromatin structure exhibits an important regulatory role on several cellular processes including transcription, replication, silencing and repair of DNA damage. To understand the role of chromatin for regulation of transcription it is important to know where nucleosomes are positioned and how positioning is achieved. Genome wide mappings of nucleosomes in *S. cerevisiae* revealed that many genes show highly positioned nucleosomes flanking a nucleosome depleted region (NDR) upstream of transcriptional start sites and downstream of stop codons [2]. These positioned nucleosomes are usually referred to as +1 and −1 for the nucleosome near the transcriptional start site and the first 5’ nucleosome, respectively. Analysis of the *CLN2* promoter showed that multiple redundant activities cooperate for the establishment of this NDR (and many others as well) and that it is essential for proper regulated expression of the gene [3]. In contrast, nucleosomes within the open reading frame of coding genes are less strictly positioned (reviewed in [4]). Here we discuss how the reorganization of chromatin structure contributes to adaptation of transcriptional programs for particular situations and requirements.

Basically there are four groups of activities which change chromatin structure during transcription: histone modifications, eviction and repositioning of histones, chromatin remodeling and histone variant exchange. Histone modifiers introduce posttranslational, covalent modifications to histone tails and thereby change the contact between DNA and histones. These modifications govern access of regulatory factors. Histone chaperones aid eviction and positioning of histones. A third class of chromatin restructuring factors are ATP dependent chromatin remodelers. These multi-subunit complexes utilize energy from ATP hydrolysis for various chromatin remodeling activities including nucleosome sliding, nucleosome displacement and the incorporation and exchange of histone variants.

**Figure 1.**
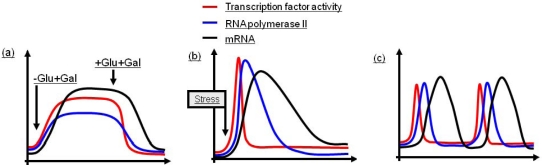
Patterns of induced transcription. (**a**) Sustained transcription is characterized by prolonged transcription factor activity depending on the induction signal resulting in moderate RNA Pol II association and sustained transcript levels; (**b**) During single pulse transcription intense transcription factor activity is followed by high RNA Pol II occupancy over a short period of time resulting in swift upregulation of transcripts and subsequent repression to basal levels; and (**c**) Oscillatory transcription appears as a circular pattern characterized by short and strong transcription factor activity as well as RNA Pol II binding. Transcripts are quickly and strongly upregulated following a harsh repression to basal levels.

Living cells need to adjust gene transcription according to diverse internal and external parameters. These signals are transmitted to the nucleus by various pathways where they trigger changes in gene expression. In principle, regulated transcription of RNA polymerase II (RNA Pol II) dependent genes can be categorized into three different patterns according to the type of inducing signals (reviewed in [5]). As shown in Figure 1, these are sustained transcription, single pulses and oscillations. Induced sustained transcription patterns switch expression of repressed genes more or less rapidly to an induced state and occur frequently during changes of metabolic programs (Figure 1A). A classic example is the regulation of the yeast *GAL1/10* locus encoding products required for galactose metabolism. *GAL1* transcription is upregulated and sustained as long as galactose is available and glucose is absent. In contrast, induced single pulse responses occur when cells encounter environmental stress such as high external osmolarity or heat shock. In these situations, transcripts are rapidly induced followed by adaptation and reduction to basal levels (Figure 1B) [6]. Finally, oscillatory expression patterns are characterized by periodic transcription and can be found in genes regulated by cell cycle and circadian rhythm (Figure 1C). In this review we focus primarily on the role of chromatin for regulation on the level of transcriptional initiation and elongation of single pulsed regulated and sustained induced genes in yeast.

## 2. Regulation of Transcriptional Activation

**Figure 2.**
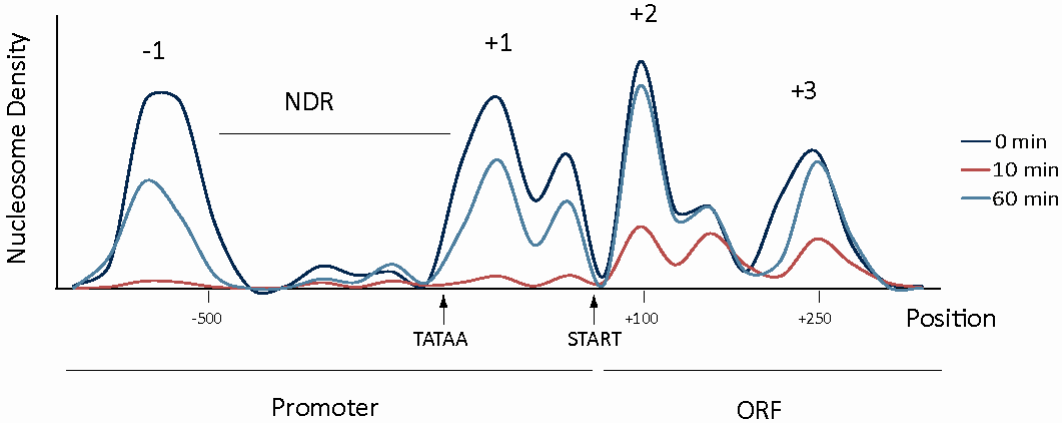
Nucleosome positions at the stress inducible *CTT1* locus: The schematic representation shows 4 positioned nucleosomes. During uninduced conditions, two (−1 and +1) are flanking the nucleosome depleted region (NDR) and belong to the promoter, whereas nucleosomes +2 and +3 are positioned at the 5’ end of the 1.7 kb long ORF (dark blue line). After induction by hyperosmolarity stress for 10 min (red line) nucleosome levels are severely depleted in the entire region, after 60 min (light blue line) chromatin structure at the ORF reaches uninduced levels whereas promoter nucleosomes are not completely reassociated.

Induction of gene transcription is triggered by the binding of transcriptional activators to specific promoter elements (upstream activation sequences) followed by recruitment of co-activators such as mediator and chromatin restructuring factors (e.g., SAGA, RSC). Thereafter, components of the general transcription machinery including TATA binding protein (TBP), general transcription factors (GTFs) and RNA Pol II assemble into the pre-initiation complex (PIC). Some loci contain preformed PICs which are paused for transcription and require additional factors for release into productive elongation [7]. However, in yeast the rate limiting step of induced transcription is activator dependent formation of the pre-initiation complex (PIC). Histones are displaced in front of elongating RNA Pol II and are rapidly reconstituted after passage. The force of transcribing RNA Pol II could be an important factor for histone displacement. Some chromatin restructuring factors are recruited to the coding regions of transcribed genes where they assist RNA Pol II during elongation. A prominent example is the histone chaperone Asf1 which co-migrates with RNA Pol II and facilitates H3–H4 eviction [8]. The physicochemical properties of DNA are almost uniform. Hence, DNA-sequence dependent processes such as transcription factor binding need to be localized on the long molecule. Chromatin structure might be important for marking promoters and transcription units much like the flag on the golf green. One important feature of chromatin is the definition of the nucleosome depleted region (NDR) immediately flanked by positioned nucleosomes at promoter regions. Nucleosomes are frequently depleted from the promoter region by certain chromatin remodeling factors (see Figure 2).

The yeast ATP dependent chromatin remodeling complexes include ATPases of the SWI2/SNF2 subfamily which are classified according to the structure of the ATPase domain. The most prominent members are SWI2, ISWI, CHD and INO80. SWI2 type ATPases have a bromodomain (SWI/SNF complex, RSC complex, Rad54) and ISWI types contain characteristic SANT and SLIDE domains (ISW1 complex, ISW2 complex). The CHD type ATPase owns a chromodomain (Chd1) whereas INO80 types are characterized by a split ATPase domain (INO80 complex, SWR1 complex) (reviewed in [9]). Each of these remodeling factors has defined activities, which are partially overlapping. The ATP dependent chromatin remodeling activity of the SWR1 complex is responsible for the exchange of canonical H2A–H2B dimers by H2A.Z–H2B [10]. The reverse reaction removes H2A.Z from chromatin and was recently identified to be INO80 dependent [11]. Two-thirds of all nucleosomes in *S. cerevisiae* contain this histone variant which is encoded by the *HTZ1* gene [12]. Genome wide maps revealed that H2A.Z is globally located at the promoter regions of inactive genes in euchromatin keeping them in a state which is poised for transcriptional activation [12–14]. Thus, H2A.Z was suggested to prevent expansion of silent chromatin into transcriptionally active euchromatin. Recent *in vitro* data suggest that SWR1 mediated deposition of H2A.Z depends on acetylation of H2A and H4 by NuA4 histone acetyltransferase (HAT) complexes [15]. NuA4 contains the Esa1 HAT subunit, shares four subunits with SWR1 and was also shown to acetylate H2A.Z after its deposition into chromatin [16]. However, the molecular mechanism by which H2A.Z contributes to regulation of transcription is still unknown. H2A.Z containing nucleosomes could act as signals for guiding activators, co-activators or general transcription factors to their appropriate positions. A recent study reported that H2A.Z also influences transcriptional elongation by promoting efficient chromatin remodeling and by stabilization of RNA Pol II elongation complexes [17].

### 2.1. Regulation of Induced, Sustained Transcription

A thoroughly studied example of a sustained transcriptional switch is the yeast bidirectional *GAL1/10* promoter targeted by the Gal4 transactivator. *GAL1* encodes a galactokinase required for one of the first steps in galactose metabolism. In the absence of galactose, Gal4 is bound by the repressor Gal80 and kept inactive. Galactose promotes binding of the regulator Gal3 to Gal80 and thus enables Gal4 to interact with co-activators (reviewed in [18]). Gal4 binding to its upstream activating sequence (UASg) is assisted by the RSC chromatin remodeling complex. RSC, assembled of 15 subunits, is closely related to the highly conserved SWI/SNF chromatin remodeling complex. Both contain an ATPase subunit comprising a DNA binding bromodomain and share two actin related proteins: Arp7 and Arp9 [19,20]. RSC is located at the UASg where it partially unwinds a single nucleosome to promote Gal4 binding [21]. The yeast SAGA complex contains 21 conserved subunits including the histone acetyltransferase (HAT) Gcn5 and is one of the first co-activators recruited by Gal4 [22]. Gcn5 acetylates specific lysine residues located on N-terminal tails of histones H3 and H4 [23]. Although SAGA was shown to be essential for PIC formation at the *GAL1/10* promoter, HAT activity is not. A strain lacking the Gcn5 HAT subunit is still able to form a functional PIC, whereas absence of one conserved subunit (Spt3) of SAGA strongly reduces PIC formation [24,25]. The SAGA complex seems to have important structural functions for PIC assembly in addition to its catalytic activity. In the presence of galactose SWI/SNF is recruited to the *GAL1/10* promoter where it is involved in the rapid removal of nucleosomes enabling Gal4 to bind additional sites. Deletion of the SWI/SNF subunit Snf2 reduces nucleosome removal from the promoter. Consequently, induction of transcription is delayed, although the overall *GAL1* transcript levels are not reduced [26]. Association of SWI/SNF partially depends on histone modifying complexes. Histone acetylation increases SWI/SNF binding and nucleosome displacement by SAGA and NuA4 complexes [26–28]. Thus, at the GAL1/10 promoter several activities interplay for fine-tuning of transcriptional regulation.

The *PHO* genes were used for pioneering studies on the influence of chromatin structure during induced transcription [29–31]. The *PHO5* gene encodes an acid phosphatase required for mobilization of phosphate. Phosphate starving conditions cause the activators Pho2 and Pho4 to bind upstream activating sequences UASp1 and UASp2 to induce *PHO5* transcription with sustained expression characteristics. At this locus SAGA, SWI/SNF and the chromatin remodeling complex INO80 are involved in transcriptional activation [32,33]. Similar to the *GAL* locus, co-activators are not essential for achieving the maximum transcript levels of *PHO5* [33]. In fact, they support promoter opening which is responsible for rapid upregulation of transcription. In contrast, the more weakly induced *PHO8* promoter is dependent on chromatin remodeling factors to a greater extent. *PHO8* activation strictly requires Snf2 and the SAGA associated HAT Gcn5. Both, the *PHO5* and *PHO8* loci require the histone chaperone Asf1 for PIC formation [34–36].

Interaction between chromatin remodeling complexes has also been observed at the *INO1* promoter. The *INO1* gene product is involved in phospholipid biosynthesis and its transcription is repressed in the presence of inositol and choline [37]. In absence of these metabolites, *INO1* promoter nucleosomes are mobilized by INO80 and SWI/SNF to activate transcription [38]. Recruitment of the SWI/SNF complex to the *INO1* promoter region depends on the presence of the Ino80 ATPase subunit [39]. Together this suggests for sustained transcribed genes that weakly regulated promoters are more susceptible to subtle changes of chromatin and thus to activity of chromatin remodeling factors, compared to strong promoters with a switch-like characteristic.

### 2.2. Regulation of Induced, Single Pulse Transcription

Yeast stress response genes are characterized by rapid and strong upregulation in response to cellular stress such as high osmolarity or heat shock followed by downregulation to almost basal levels. This pattern of transcription is referred to as “single pulse transcription” (Figure 1B). One of the best studied conditions is increased extracellular osmolarity which is sensed and transmitted by the mitogen activated protein (MAP) kinase cascade called hyperosmolarity glycerol response (HOG) pathway. Activation of the Map kinase Hog1 leads to its translocation into the nucleus and subsequent activation of genes via certain transcription factors. The transcription factor Smp1 is phosphorylated and activated by Hog1. Hog1 also activates the transcription factor Hot1 by phosphorylation. Importantly, Hot1 dependent genes show interactions of Hog1 with components of the PIC such as mediator and RNA Pol II and directly associate to chromatin [40,41]. Hog1 binds to the stress transcription factors Msn2 and Msn4 and becomes recruited to chromatin but does not phosphorylate them. Chromatin remodeling is then facilitated by Hog1 induced recruitment of RSC to the activated stress genes. Furthermore, Hog1 directs the Rpd3L deacetylase complex to promoters which is required for the induction of environmental stress response genes [42–44]. The histone deacetylase Rpd3 is a subunit of two complexes: Rpd3L and Rpd3S. While Rpd3L has an activating role for stress gene transcription, the repressive roles which have been associated with both complexes will be discussed later. These observations demonstrate a much more general role of MAP kinases for transcriptional regulation. Interaction between a stress activated protein kinase and transcription factors was also confirmed in higher eukaryotes [45]. At heat shock genes the chromatin remodelers RSC, SWI/SNF and ISWI are involved in transcriptional activation. Preloading of the transcriptional activator Hsf1 requires ISWI and RSC complexes indicating that at heat shock genes, similar to the *GAL1/10* promoter, chromatin remodeling occurs prior to activator binding [46].

Taken together, induced transcription strongly depends on activator dependent recruitment of co-activators to form a functional PIC. These co-activators promote acetylation and deacetylation of histone tails, incorporation of histone variants and ATP dependent chromatin remodeling. In case of the strongly induced *GAL1* and *PHO5* promoters, histone acetylation and nucleosome remodeling are not essential for PIC formation. These activities have a more important role in facilitating swift upregulation of transcript levels by removal of promoter nucleosomes. During elongation several chromatin restructuring factors travel with elongating RNA Pol II and assist in removing nucleosomes and restoration of canonical chromatin.

## 3. Repressive Role of Chromatin

Gene activation and elongation correlate with intense displacement of nucleosomes at promoter and transcribed regions (see Figure 2) [8]. Disturbed chromatin structures need to be restored to facilitate efficient repression of target genes and to avoid transcription from cryptic initiation sites [8,47,48]. The events that promote restoration are promoter closing as well as repositioning of displaced nucleosomes in coding regions. Additionally, deacetylation of histones plays an important role in this respect resulting in tight packaging of chromatin. Similar to activation, transcriptional repression requires several chromatin restructuring factors such as histone chaperones, chromatin remodelers and histone modifiers. Repressive activities act redundantly at many gene loci. Strains lacking components of the ATP-dependent chromatin remodeling complex ISW1, the H4/H2A acetyltransferase complex NuA4 and the SWR1 complex have globally changed transcript profiles including upregulation of many stress genes [49]. In addition, the ATP-dependent chromatin remodeling factor Isw2 was correlated with gene repression by positioning of nucleosomes to unfavorable poly dA-dT DNA sequences in promoter regions. At the *POT1* promoter, absence of Isw2 causes a specific promoter nucleosome to changes its position to a more favorable sequence resulting in activation of the gene [50].

As already mentioned, the histone deacetylase Rpd3 is a subunit of two complexes: Rpd3L and Rpd3S. Both have been associated with transcriptional repression. The Rpd3L large histone deacetylase complex (HDAC) is recruited to the *INO1* promoter where it removes H4 K5 acetylation under non-induced conditions [51]. Rpd3S represses transcription of target genes and initiation from cryptic sites by deacetylation within coding regions [52,53]. These observations suggest that deacetylation of histones in promoter regions suppresses transcription prior to induction, whereas deacetylation of histones in coding regions supports downregulation and avoids initiation from cryptic sites. At the level of initiation HDACs create compacted, hypoacetylated regions that suppress PIC formation at promoter regions. During elongation these modifiers as well as elongation factors associate to coding regions to avoid cryptic transcription [47,48].

Histone chaperones also function in the repression of induced genes. They are predominantly associated with elongating RNA Pol II and restore canonical chromatin structure. Asf1 and Spt6 have been intensively studied for their activities during transcriptional regulation. Asf1 interacts with H3–H4 dimers and is associated to promoters and coding regions of active genes. In case of the inducible *GAL1/10* system Asf1 is necessary for histone eviction and positioning and thus is involved in activation and repression [8]. In case of some stress induced genes Asf1 has a strictly repressive role and is not required for activation [54].

The histone chaperone Spt6 interacts directly with phosphorylated Serine2 of RNA Pol II CTD [55]. Spt6 has been described as a repressive factor with a varying role dependent on the particular gene. The mechanism of how Spt6 influences transcription is not known yet. At highly induced genes absence of Spt6 leads to loss of open reading frame nucleosomes and in case of the serine-inducible *CHA1* locus to the delocalization of the +1 nucleosome [56]. Spt6 is required for repression of strongly induced stress genes [54]. In addition to Spt6, the INO80 complex is involved in transcriptional repression of stress genes. Strains lacking the INO80 subunit Arp8 show higher and prolonged transcript levels of stress genes. This effect was observed for several single pulse genes induced by high osmolarity, heat shock or copper stress. The hyperinduction coincides with a delay in restoration of chromatin structure along promoter and open reading frame regions and genetic data suggest that Spt6 and Arp8 are required for the same function [54]. The unique expression kinetics of stress induced genes might cause disturbance of chromatin leading to generation of a signal attracting INO80. Transcriptional activation by the stress responsive transcription factor Msn2 is sufficient to attract INO80. In DNA repair, the INO80 complex is recruited to double strand breaks via the Nhp10 subunit which interacts with S129 phosphorylated H2A (gamma-H2AX) [57]. This mechanism does not function for the recruitment to transcription sites, suggesting an alternative way directing chromatin remodelers to their appropriate positions [54].

Basically there are two different activities of INO80 described which could contribute to the observed transcriptional effects. INO80 is able to slide nucleosomes along DNA without dissociation and able to reduce the distance between nucleosomes on an artificial DNA construct [58]. In addition, INO80 could also contribute to the regulation of gene expression by exchanging histone variants for canonical histones. The INO80 complex triggers replacement of H2A.Z–H2B dimers by H2A–H2B and thus is involved in removal of H2A.Z [11]. A recent publication claims that H2A.Z supports transcriptional elongation by facilitating the correct assembly and modification status of RNA Pol II elongation complex [17]. This would favor a model where the INO80 complex removes H2A.Z from coding regions, thereby reducing elongation. Presumably both, nucleosome sliding and H2A.Z removal could play together for the downregulation of stress genes by the INO80 complex.

## 4. Conclusions

In summary, activation and repression of induced genes are influenced by chromatin restructuring factors. The factors involved as well as the importance of these factors for transcriptional regulation varies for different loci. Some are essential for PIC formation and thus for activation whereas others are required for fine-tuning of transcript levels. Multiple studies have shown that transcriptional activation and repression are regulated at the level of initiation and elongation. Although functions of these factors have been extensively characterized, many of the signals which direct them to distinct loci remain unclear. It is speculated that characteristic induction kinetics may also be a signal to attract certain chromatin restructuring factors.
